# Magmas Overexpression Inhibits Staurosporine Induced Apoptosis in Rat Pituitary Adenoma Cell Lines

**DOI:** 10.1371/journal.pone.0075194

**Published:** 2013-09-17

**Authors:** Federico Tagliati, Teresa Gagliano, Erica Gentilin, Mariella Minoia, Daniela Molè, Ettore C. delgi Uberti, Maria Chiara Zatelli

**Affiliations:** 1 Section of Endocrinology, Department of Medical Sciences, University of Ferrara, Ferrara, Italy; 2 Laboratorio in rete del Tecnopolo “Tecnologie delle terapie avanzate” (LTTA) of the University of Ferrara, Ferrara, Italy; IISER-TVM, India

## Abstract

Magmas is a nuclear gene that encodes for the mitochondrial import inner membrane translocase subunit Tim16. Magmas is overexpressed in the majority of human pituitary adenomas and in a mouse ACTH-secreting pituitary adenoma cell line. Here we report that Magmas is highly expressed in two out of four rat pituitary adenoma cell lines and its expression levels inversely correlate to the extent of cellular response to staurosporine in terms of apoptosis activation and cell viability. Magmas over-expression in rat GH/PRL-secreting pituitary adenoma GH4C1 cells leads to an increase in cell viability and to a reduction in staurosporine-induced apoptosis and DNA fragmentation, in parallel with the increase in Magmas protein expression. These results indicate that Magmas plays a pivotal role in response to pro-apoptotic stimuli and confirm and extend the finding that Magmas protects pituitary cells from staurosporine-induced apoptosis, suggesting its possible involvement in pituitary adenoma development.

## Introduction

Magmas (**m**itochondria-**a**ssociated protein involved in **g**ranulocyte-**ma**crophage colony-stimulating factor **s**ignal transduction) is a nuclear gene that encodes for the mitochondrial import inner membrane translocase subunit Tim16. It was first reported as a granulocyte-macrophage colony-stimulating factor (GM-CSF) induced protein in the murine myeloid cell line PGMD1, where Magmas mediates GM-CSF proliferative effect [1]. Magmas is highly conserved and ubiquitously expressed in all mammalian cells [2,3], with a inter-species overlapping activity, suggesting that it is essential for cell viability [3]. In silico studies indicate that, in fungi, Magmas can be transduced in two different protein isoforms. One isoform contains a mitochondria target peptide (mTP) which results in Magmas targeting to the mitochondria; the alternative isoform does not contain the mTP motif, resulting in a cytoplasmic protein localization [3]. Similarly, in plants Magmas protein displays the mTP motif or a different signal peptide, with consequent different cellular localization. In *Saccharomyces Cerevisiae*, Tim16 ortholog is represented by the presequence translocase associated protein import motor (PAM) 16, which has been shown to be important for protein translocation across the mitochondrial inner membrane [4,5]. PAM is formed by five subunits, represented by the matrix heat shock protein 70 (mtHsp70) and four cochaperones: Mge1, Tim44, Pam18 and Pam16. All together these proteins form the motor of the translocase of inner membrane (TIM) complex, which drives proteins from the mitochondrial intermembrane space to the matrix across the inner membrane [6,7]. Pam16 and Pam18 form a heterodimer, the integrity of which is crucial for cell growth and mitochondrial protein import [8,9].

The role of Magmas in cell growth is still unclear. It has been reported that its expression levels are increased in a significant proportion of human prostate cancers, independently of mitochondria content [10]. In addition, we previously reported that Magmas mRNA is over expressed in two ACTH-secreting mouse pituitary adenoma cell lines as compared to normal mouse pituitary, as well as in 47 out of 64 human pituitary adenomas compared to normal human pituitary [11]. We also found that Magmas silencing sensitizes to pro-apoptotic stimuli and induces a G0/G1 accumulation [11]. The aims of our study are to verify whether Magmas protective effects towards apoptotic stimuli are restricted to the ACTH-secreting pituitary adenoma cell line and to elucidate Magmas protein function in regulating cell survival by exploring the mechanisms underlying its protective effects, by over-expressing this gene in rat pituitary cell lines.

## Materials and Methods

### Materials

All reagents, if not otherwise specified, were purchased from Sigma-Aldrich (Milano, Italy).

### Cell culture

GH4C1, GH3, GH1, and MMQ rat pituitary adenoma cell lines were obtained from ATCC (American Type Culture Collection, Manassas, VA, USA). Growth hormone (GH) and prolactin (PRL) secreting GH3 and GH1 adhesion cell lines and the PRL secreting MMQ suspension cell line were cultured in the Ham’s F12K medium enriched with 15% horse serum (HS) and 2.5% fetal bovine serum (FBS), as previously described [12]. GH/PRL secreting GH4C1 adhesion cell line was maintained in Ham’s F-10 Nutrient enriched with 15% HS and 2.5% FBS.

### RNA extraction

Total RNA was extracted using TRIzol reagent following manufacturer’s instructions (Life Technologies, Milano, Italy), as previously described [13,14]. A RNA pool from normal rats (Swiss Webster rats) was purchased from Ambion (Life Technologies). RNA integrity was evaluated with the Experion automated electrophoresis system (Bio-Rad Laboratories, Milano, Italy). Only RNA samples with a 28S/18S rRNA ratio >1.6 and a RNA quality indicator >9 were used in further experiments. All RNA samples were subjected to DNase I treatment, as previously described [11].

### Reverse Transcription (RT) and qPCR

Reverse Transcription (RT) and quantitative real time PCR (qPCR) were performed as previously described [11,15]. Briefly, qPCR to assess Magmas expression was performed using TaqMan gene expression assay (Rn01424695_g1) (Life Technologies). Samples were run in triplicate on Applied Biosystems 7700 ABI Prism thermal cycler and analyzed with the SDS 1.9 software (Life Technologies). To ensure the fidelity of mRNA extraction and RT, target gene signals from all samples were normalized against five different reference genes: 18S rRNA, glyceraldehyde-3-phosphate dehydrogenase, β-actin, ribosomal protein large P0, and cyclophilin. Primers and probes for all reference gene mRNAs were commercially available (TaqMan endogenous control; Life Technologies). Calculations to estimate the expression stability and the pair-wise variation were performed with the freely available GeNorm program. The 18S rRNA showed the most stable expression between samples, and therefore was used as reference gene.

### Cell viability

The effect of Staurosporine, a well known apoptotic stimulus, on cell viability was assessed by measuring cellular ATP levels using the ATPLite luminescence-based assay (PerkinElmer, Waltham, MA, USA) following manufacturer’s instructions and reading the plate with the EnVision Multilabel Reader (PerkinElmer) as previously described [16,17]. Briefly, 2 x 10^4^ cells/ well were plated in optically clear 96-well black plates (Costar #3904, Corning, NY, USA) and incubated overnight at 37 °C. Staurosporine was diluted in culture medium immediately before use. All samples were assayed in quadruplicate in three independent experiments and are reported as the mean ± SE.

### Plasmids and Transfection

Magmas cDNA coding sequence was amplified using the following primers: forward 5’-GTC GAC GGT ACC GCG GGC CCA TGG CCA AGT ACC TGG CCC AGA -3’ and reverse 5’- GAC CGG TGG ATC CCG GGC CCC GTT TTG GGC GTC TGC CCT TTC -3’. The resulting fragment was subcloned in-frame in the pPTunerC Vector C-terminal by ApaI digestion following manufactures instructions (Clontech, Mountain View, CA, USA). For all constructs the proper gene cloning was confirmed by sequencing, performed as previously described [18]. The employed vector encodes for a fusion protein composed by Magmas fused in 3’ with a 12 kDa FKBP-based destabilization domain (DD-C) that has been optimized for use as a C-terminal tag [19]. This domain, located just downstream of the multiple cloning site, causes the rapid degradation of any protein to which it is fused. Once expressed, DD-C-tagged protein amount can be rapidly increased by the addition of the Shield1 stabilizing ligand to the medium. Shield1 is a membrane permeant molecule that binds to the DD-C tag, 'shielding' the fusion protein from proteasomal degradation. Transfections were carried out as described previously [20].

### Isolation of mitochondrial and cytosolic fractions

For Western blot analysis, mitochondrial and cytosolic protein fractions were separated using the Qproteome Mitochondria Isolation Kit following manufacturer’s instructions (Qiagen, Milano, IT). Proteins were then isolated by adding RIPA Buffer (Euroclone, Milan, IT) in ice for 30 minutes and then centrifuging for 10 minutes. The protein containing supernatant was then transferred to a new tube and protein concentration was measured by BCA Protein Assay Reagent Kit (Pierce, Rockford, IL, USA), as previously described [21,22].

### Western blot analysis

Total proteins from pituitary adenoma rat cell lines and from a pool of five normal rat pituitaries were isolated as described above. Forty µg of proteins were fractionated on 14% SDS-PAGE for Magmas protein or on 10% SDS-PAGE for all the other investigated proteins, as previously described [21], and transferred by electrophoresis to Nitrocellulose Transfer Membrane (PROTRAN, Dassel, Germany). Membranes were incubated with the following primary antibodies: cytochrome c (1:1000) (cat. #11940), Bax (1:1000) (cat. #2772), caspase 3 (1:1000) (cat. #9665), caspase 9 (1:1000) (cat. #9508), Bcl2 (1:1200) (cat. #2870), tubulin (1:1000) (cat. #2128), actin (1:2000) (cat. #4970) all form Cell Signalling (Beverly, MA, USA), TOMM22 (1:2000) (cat. ab134274) (AbCAM, Cambridge, UK) and Magmas (PRIMM, Milano, Italy) at 1:1000. Horseradish peroxidase-conjugated secondary antibody IgG (Dako, Milano, Italy) (cat. P0448) was used at 1:5000 and binding was revealed using enhanced chemiluminescence (Pierce).

### Cell count

Cells were plated at 2 x 10^5^ cells/well, transfected and treated with 100 nM Staurosporine. Cell number was assessed 12 h, 24 h, 48 h and 72 h by using the CyFlow Space cytometer (Partec, Italy Srl., Carate Brianza, Italy) at least three times.

### Caspase activity and DNA fragmentation analysis

Caspase activity was measured using Caspase-Glo 3/7 assay (Promega, Milano, Italy), as previously described [23] on the EnVIsion Multilabel Counter (PerkinElmer, Monza, Italia). Briefly, cells were seeded at 2 × 10^4^ cells/well in 96-well white-walled plates, and treated with the indicated compounds for 48 h. Results are expressed as mean value ± SE relative light units vs. vehicle-treated control cells. DNA fragmentation analysis was performed as previously described [11].

### Cell cycle analysis

Cell cycle phase distribution analysis was performed by flow cytometry after DNA staining, as previously reported [24]. Briefly, 3 x 10^6^ cells were collected in GM solution, fixed dropwise with 70% ice-cold ethanol, washed twice at room temperature with PBS (Phosphate Buffered Saline), resuspended in extraction buffer (CyStain PI Absolute T, Partec, Italy Srl), and incubated at room temperature for 15 minutes. Staining solution containing Propidium Iodide (PI) and RNase was prepared as reported in the manufacturer instruction and added to cell extracts, followed by incubation at room temperature over night. DNA PI-associated fluorescence in all cells was measured by CyFlow Space cytometer (Partec, Italy Srl). A total of 20.000 events were captured for each treatment and analyzed with FlowMax software (Partec, Italy Srl).

### Apoptosis Assays

Cells were plated at 2 x 10^5^ cells/well into 4 cm^2^ wells 24 hours prior to transfection without (mock) or with pPTunerC-Magmas-DD in the presence or in the absence of 200 nM Shield1 for 12 hours. Cells were then treated with or without 100 nM Staurosporine and maintained at 37°C for 24 hours. Cells were harvested by trypsinization, washed twice with PBS and suspended in ice cold annexin V buffer (10 mM HEPES-KOH pH 7.4, 140 mM NaCl and 2.5 mM CaCl_2_) with FITC-conjugated annexin V and incubated on ice for 15 min. Cells were then co-stained with 50 µg/ml propidium iodide in PBS and analyzed using CyFlow Space cytometer (Partec, Italy Srl) and FlowMax software (Partec, Italy Srl).

### Mitochondrial membrane potential (MMP) assay

GH4C1 transfected with pPTunerC-Magmas-DD were treated without or with 100 nM Staurosporine and MMP was evaluated by employing the JC-1 Mitochondrial membrane potential assay kit following manufacturer instruction (Cayman Europe, Estonia). Briefly, MMP was determined by incubating the cells with JC-1 dye at 37°C for 30 min. The fluorescence intensity was measured using the EnVIsion Multilabel Counter (PerkinElmer), and the MMP was expressed as the ratio between 590 nm (red) and 529 nm (green) emission.

### Statistical analysis

Results of cell viability and caspase activation experiments are expressed as the mean ± SE [25]. A preliminary analysis was carried out to determine whether the datasets conformed to a normal distribution, and a computation of homogeneity of variance was performed using Bartlett’s test. The results were compared within each group and between groups using ANOVA. If the F values were significant (P<0.05), Student’s paired or unpaired t-test was used to evaluate individual differences between means. P values <0.05 were considered significant. For all the other experiments, results are expressed as the mean ± SE among at least three replicates. Student’s paired or unpaired t-test was used to evaluate individual differences between means and P values <0.05 were considered significant.

## Results

### Magmas expression in rat pituitary adenoma cell lines

**Figure 1:**
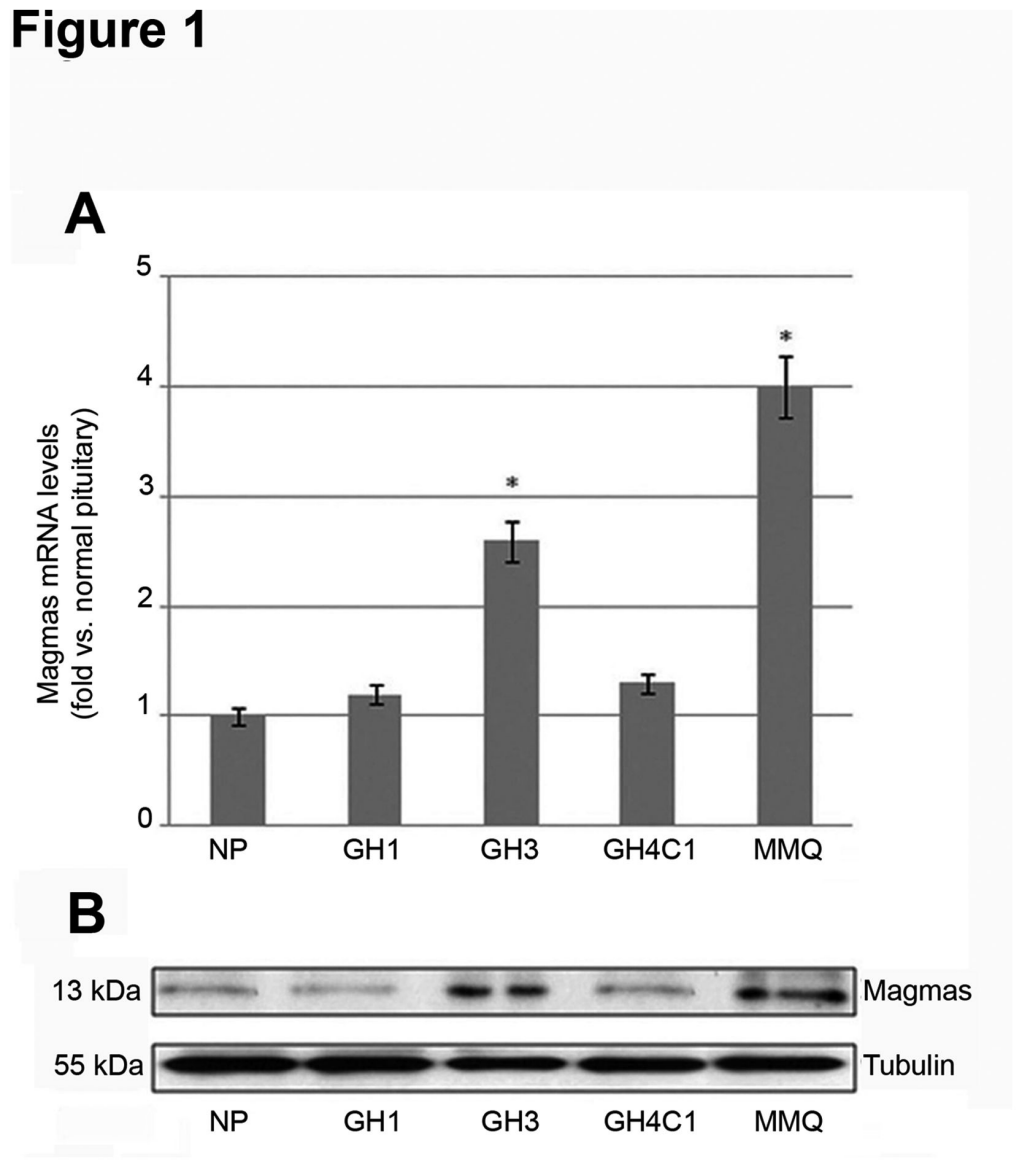
Magmas expression in rat pituitary cell lines. (A) Expression of rat Magmas gene in GH1, GH3, GH4C1 and MMQ cell lines was determined by RT-qPCR and normalized to 18S rRNA as reference gene. Data are presented as fold change of mRNA levels of target gene (mean ± SE) in the cell lines vs. normal pituitary pool (NP), as detailed in Materials and Methods. *P < 0.05. (B) Expression of rat Magmas protein in GH3, GH4C1 and MMQ cell lines was determined by Western blot analysis and normalized to tubulin as housekeeping gene.

To define Magmas expression in rat pituitary adenoma cell lines we assessed Magmas mRNA levels in 4 rat pituitary adenoma cell lines compared with a pool of normal rat pituitaries by RT-qPCR. Magmas mRNA levels were 2.6- and 4-fold higher in GH3 and MMQ cells, respectively, as compared with the rat normal pituitary tissue pool (Figure 1A). Magmas mRNA levels in GH1 and GH4C1 cells were similar to those detected in the rat normal pituitary tissue pool (Figure 1A). Comparable results were obtained by Western blot analysis (Figure 1B).

### Rat pituitary cell lines display different Staurosporine-induced apoptosis

**Figure 2:**
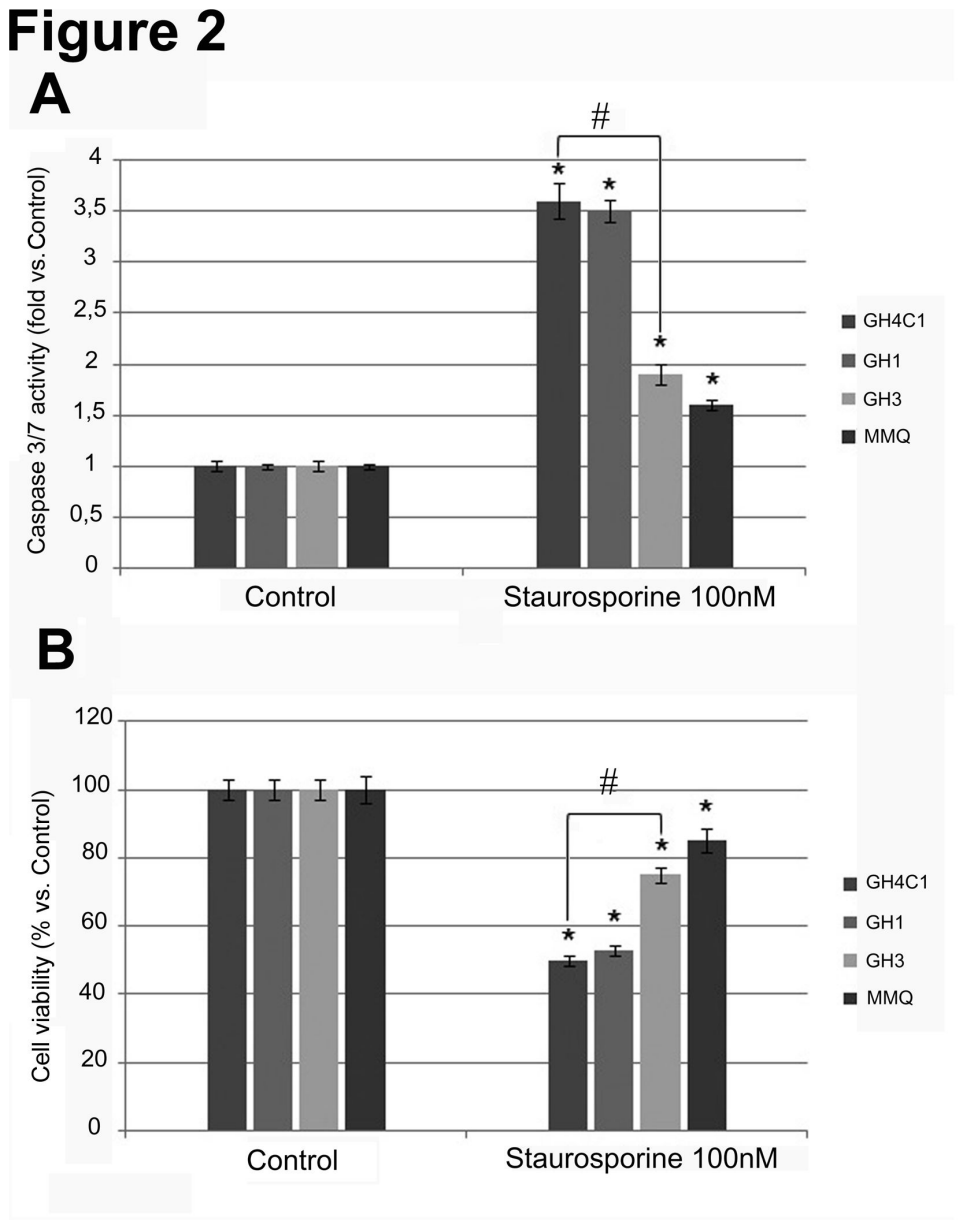
Effects of Staurosporine on caspase 3/7 activation and cell viability in GH1, GH3, GH4C1 and MMQ cell lines. All cell lines were incubated in 96-well plates for 48 h in culture medium supplemented with 100 nM Staurosporine, and control cells were treated with vehicle solution. (A) Caspase 3/7 activation was assessed as described in the Materials and Methods section. Data were evaluated in at least six independent experiments with eight replicates each and are expressed as the mean value ± SE fold induction vs. control cells. *P< 0.05 vs. control cells. # P < 0.05 vs. GH4C1-treated cells. (B) Cell viability was assessed as described in the Materials and Methods section. Data were evaluated in at least six independent experiments with eight replicates each and are expressed as the mean value ± SE percent ATP increase vs. control cells. * P < 0.05 vs. control cells. # P < 0.05 vs. GH4C1-treated cells.

To investigate the role of Magmas in apoptosis, we evaluated Staurosporine-induced apoptosis in four different cell lines: the GH4C1 and GH1 cells, that express Magmas at levels comparable to those of normal pituitary, and the GH3 and MMQ cells, that express Magmas at higher levels as compared to normal pituitary. Cells were incubated in culture medium for 48 h without or with 50-400 nM Staurosporine. As shown in Figure 2A, 100 nM Staurosporine significantly (P<0.05) induced Caspase 3/7 activity to a greater extent in GH4C1 cells (3.6-fold vs. control cells) and GH1 cells (3.5-fold vs. control cells) as compared to GH3 cells (1.8-fold vs. control cells; P<0.05 vs. GH4C1 cells) and MMQ cells (1.6-fold vs. control cells). To verify whether the apoptotic mechanism associates with a reduction in cell viability, the latter was evaluated by measuring cell viability in the same conditions. As shown in Figure 2B, Staurosporine significantly (P<0.05) reduced ATP levels to a greater extent in GH4C1 cells (-48% vs. control cells) and GH1 cells (-45% vs. control cells) as compared to GH3 cells (-24% vs. control cells; P<0.05 vs. GH4C1 cells) and MMQ cells (-17% vs. control cells). These results indicate that Magmas levels inversely correlate to the extent of cellular response to pro-apoptotic stimuli.

### Magmas over-expression in GH4C1 cells

**Figure 3:**
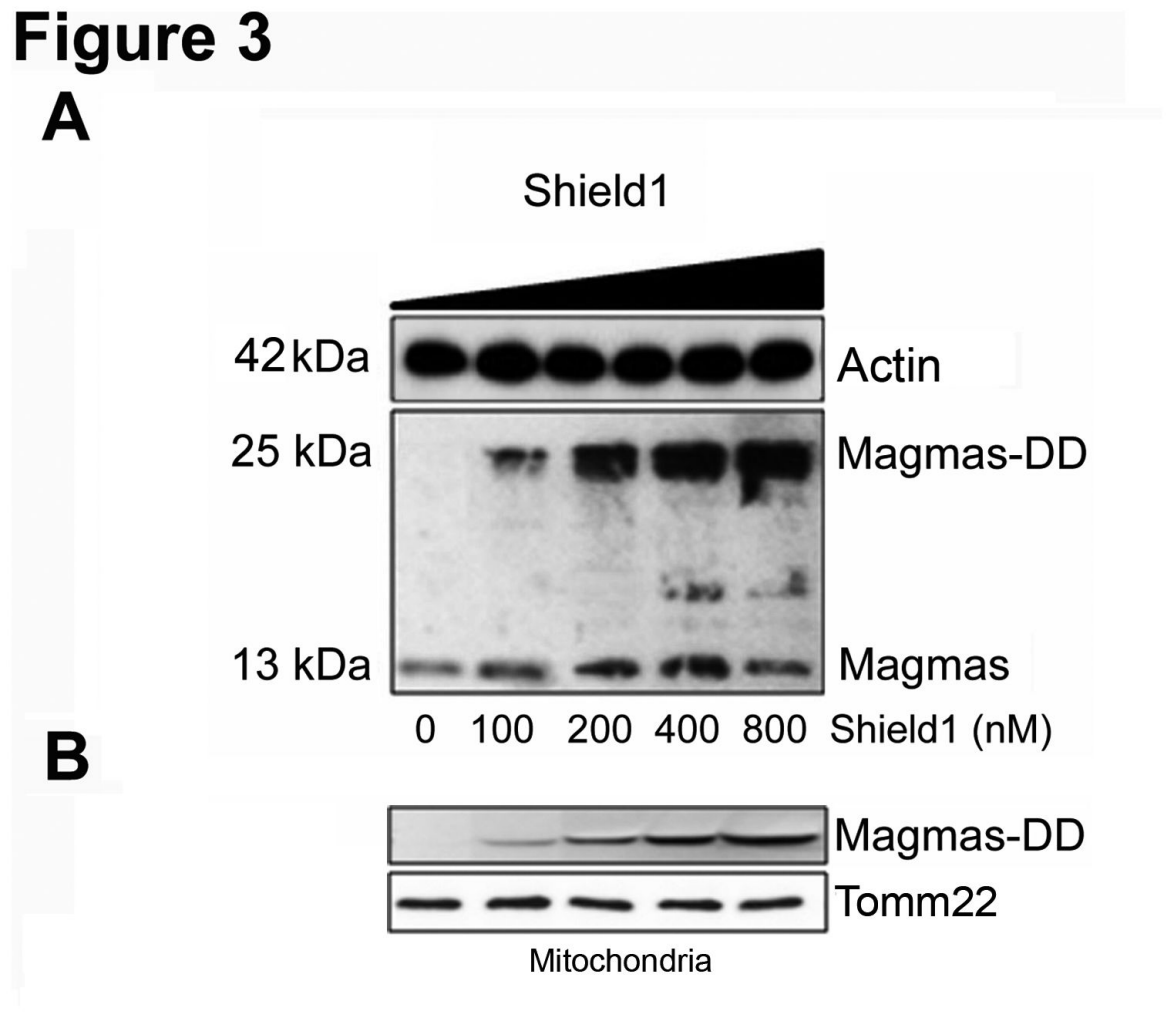
Magmas-DD expression in GH4C1 cells. (A) GH4C1 cells were transfected with the pPTunerC Magmas-DD vector and treated without or with increasing concentrations of Shield1 (100-800 nM) for 12 h. Western blot analysis shows the transfected Magmas-DD protein (25 kDa), endogenous Magmas (13 kDa), as well as the internal control, actin (42 kDa). (B) GH4C1 cells were transfected with the pPTunerC Magmas-DD vector and treated without or with increasing concentrations of Shield1 (100-800 nM) for 12 h. Western blot analysis on mitochondria extracts shows the transfected Magmas-DD protein (25 kDa) as well as the internal control, Tomm (17 kDa).

We previously demonstrated that Magmas silencing sensitizes mouse pituitary cells to apoptotic stimuli [11]. We therefore aimed at confirming the protective effect of Magmas towards pro-apoptotic stimuli also in rat pituitary cell lines and investigated the mechanism underlying this effect. To this purpose, we over-expressed Magmas in GH4C1 cells that do not over-express the gene since they display basal Magmas levels similar to those observed in normal rat pituitary. We employed an expression vector that allows precise regulation of the amount of protein by adding or removing the Shield1 stabilizing ligand to the culture medium. To verify whether Magmas fusion protein (Magmas-DD) was indeed expressed in GH4C1 cells transfected with the pPTunerC Magmas-DD vector (GH4C1-M-DD cells), Western blot analysis was performed at different time points (from 2 to 96 h) and with increasing Shield1 concentrations. Recombinant Magmas protein becomes stable after incubation with Shield 1 for 12 hours and up to 96 hours. As shown in Figure 3A, medium supplementation with increasing Shield1 concentrations determined a dose-dependent increase in Magmas-DD protein expression, which overreached endogenous Magmas protein levels, that was almost steady throughout the experiment.

To verify the mitochondrial localization of the Magmas-DD fusion protein, we performed Western blot analysis on mitochondrial extracts. As shown in Figure 3B medium supplementation with increasing Shield1 concentrations determined a dose-dependent increase in Magmas-DD protein in the mitochondrial extracts.

### Magmas over-expression promotes cell viability and counteracts Staurosporine- induced apoptosis

Preliminary studies in Magmas silenced GH4C1 cells showed overlapping results to those already reported in a mouse pituitary adenoma cell line [11] (data not shown).

**Figure 4:**
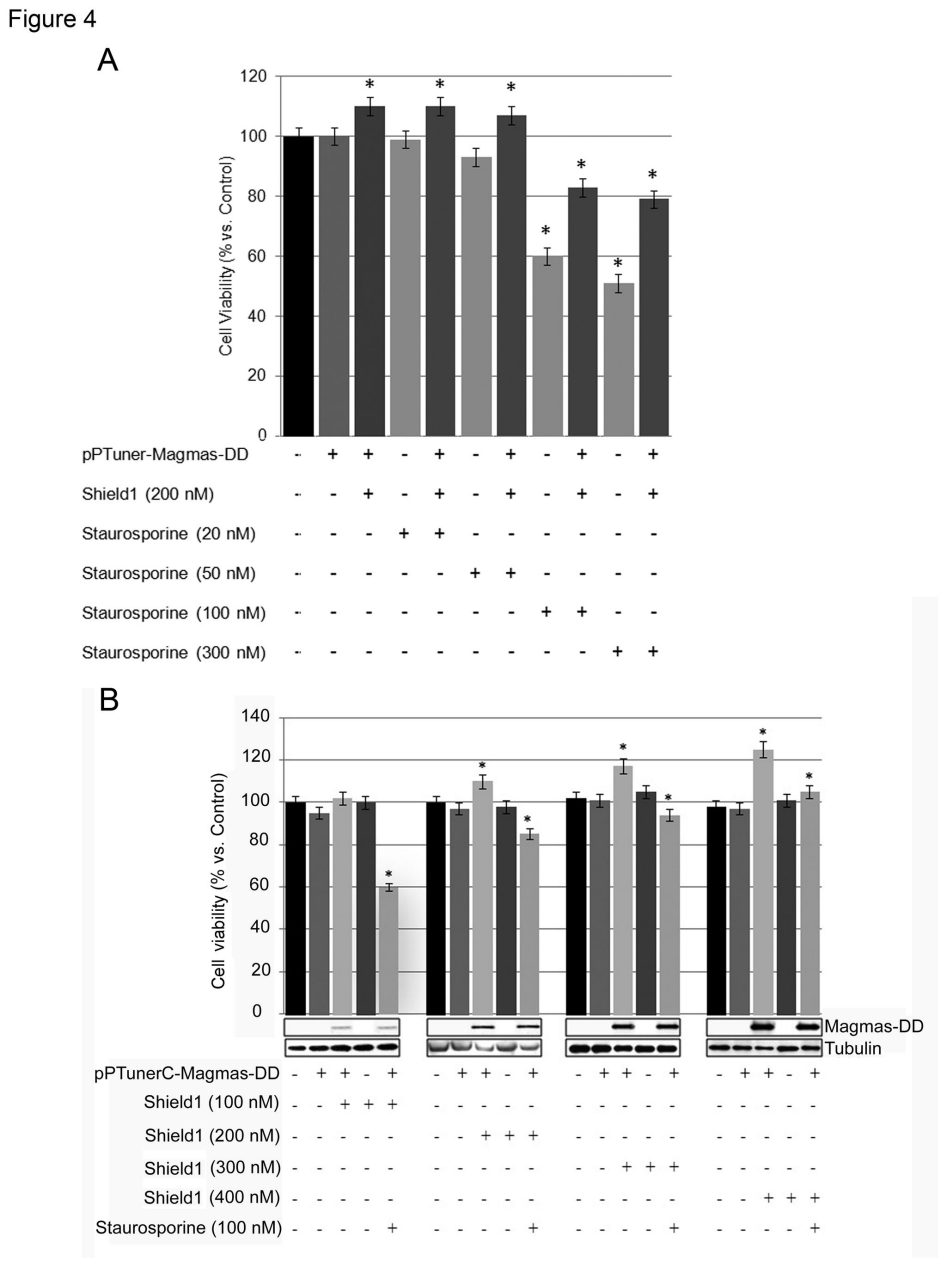
Magmas over-expression increases cell viability and counteracts Staurosporine effects in GH4C1-M-DD cells. (A) GH4C1 were transfected or not with the pPTunerC Magmas- DD vector and then incubated in 96-well plates for 48 h in culture medium in the absence or in the presence of 200 nM Shield1 for 12 h before adding Staurosporine at increasing concentrations (from 20 to 300 nM). Data were evaluated in at least three independent experiments with four replicates each and are expressed as the mean value ± SE percent cell viability vs. control cells. *P < 0.05 vs. control cells. #P<0.05 vs. GH4C1 cells treated with Staurosporine. (B) GH4C1 were transfected or not with the pPTunerC Magmas- DD vector and then incubated in 96-well plates for 48 h in culture medium supplemented in the absence or in the presence of increasing Shield1 concentrations(from 100 nM to 400 nM) for 12 h before adding or not 100 nM Staurosporine. Cell viability were then assessed as described in the Materials and Methods section. Data were evaluated in at least three independent experiments with four replicates each and are expressed as the mean value ± SE percent cell viability vs. control cells. *P < 0.05 vs. control cells. #P<0.05 vs. GH4C1 cells treated with Staurosporine. Western blot analysis (middle panel) shows Magmas-DD protein expression levels, as well as the internal control, tubulin.

To further evaluate the influence of Magmas on rat pituitary cell viability, we transfected GH4C1 cells with the pPTunerC Magmas-DD vector, encoding for the Magmas-DD fusion protein, and then evaluated cell viability and cell number. Figure 4A shows a Staurosporine titration assay in GH4C1 cells transfected or not with pPTunerC Magmas-DD. In GH4C1-M-DD cells, in the absence of Shield1 viability significantly decreased when Staurosporine was added to a final concentration of 100-300 nM (-40 and -55% vs. control, respectively; p<0.05), while no significant effect was seen at lower concentrations. On the other hand, in GH4C1-M-DD cells in the presence of Shield 1 cell viability increased by ~10% despite co-incubation with Staurosporine 20-50 nM (p<0.05). However, in the presence of Staurosporine 100 and 300 nM viability significantly decreased by ~20% (p<0.05 vs. control). The reduction in cell viability under Staurosporine 100 and 300 nM was greater in GH4C1-M-DD cells in the absence of Shield1 as compared to that observed in the presence of Shield1 (-20 and -30%, respectively; p<0.05).

As shown in Figure 4B, pPTunerC Magmas-DD transfection alone did not significantly modify GH4C1 cell viability. Similar effects were observed when GH4C1 cells were treated with increasing concentrations of Shield1 (100-400 nM). On the other hand, in GH4C1-M-DD cells, viability significantly increased when Shield1 was added to a final concentration of 200 nM or greater. The observed increase in cell viability paralleled with the increase in Magmas-DD protein expression, indicating that Magmas over-expression specifically causes an increase in GH4C1 cell viability.

Treatment with 100 nM Staurosporine significantly reduced cell viability (-40%; P<0.05 vs. control) in GH4C1-M-DD cells, in the presence of the lowest employed concentration of Shield1 (100 nM). However, the inhibitory effects of Staurosporine on cell viability were counteracted by co-treatment with higher concentrations of Shield1. The observed reduction in the inhibitory effects of Staurosporine on cell viability paralleled with the increase in Magmas-DD expression, indicating that Magmas over-expression blunts the inhibitory effects of Staurosporine on cell viability.

**Figure 5:**
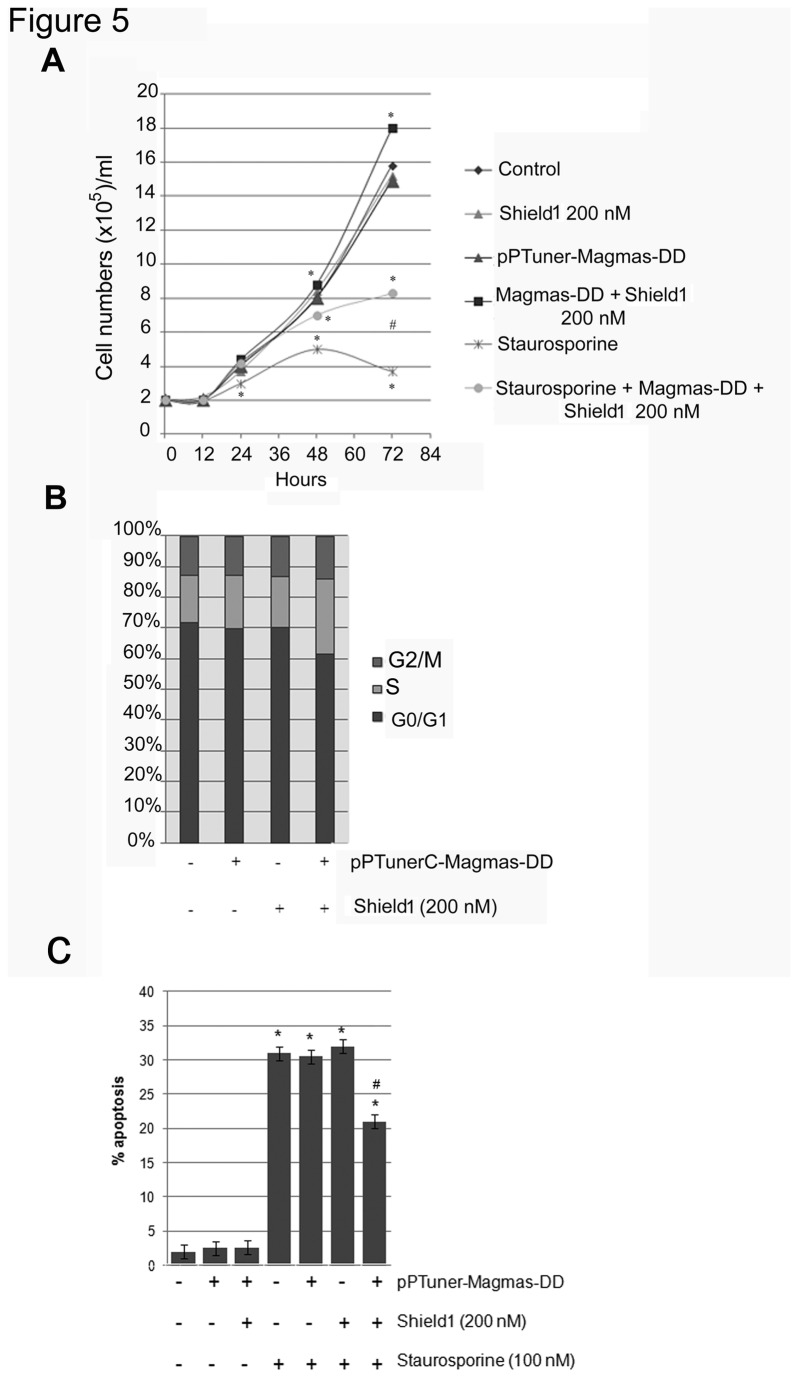
Magmas over-expression increases cell number and counteracts Staurosporine-induced apoptosis in GH4C1-M-DD cells. (A) GH4C1 were transfected or not with the pPTunerC Magmas- DD vector and then incubated in 96-well plates for 12, 24, 48 and 72 h in culture medium supplemented with or without 100 nM Staurosporine, in the absence or in the presence of 200 nM Shield1 concentrations. Cell count was then assessed as described in the Materials and Methods section. Data were evaluated in at least three independent experiments with four replicates each and are expressed as the mean value ± SE cell number/ml vs. control cells. *P < 0.05 vs. control cells. (B) GH4C1 were transfected or not with the pPTunerC Magmas-DD vector. Cell-cycle analysis was performed after treatment with or without 200 nM Shield 1. The graph shows representative data of three independent experiments, which were repeated three times. (C) GH4C1 were transfected or not with the pPTunerC Magmas- DD vector, incubated 24 h in culture medium supplemented with or without 100 nM Staurosporine, and analyzed for apoptosis after 24 hours. Cells were stained with FITC-conjugated annexin V and propidium iodide. Data were evaluated in at least three independent experiments with three replicates each and are expressed as % apoptosis. *P < 0.05 vs. control cells; #P<0.05 vs. GH4C1 cells treated with Staurosporine.

As shown in Figure 5A, pPTunerC Magmas-DD transfection alone did not significantly modify GH4C1 cell number. A similar effect was observed when GH4C1 cells were treated with 200 nM Shield1. On the other hand, Staurosporine significantly reduced GH4C1 cell number (-45%; P<0.05 vs. control). In addition, the number of GH4C1-M-DD cells significantly increased (+12.5%; P<0.05 vs. control) in the presence of 200 nM Shield1, that counteracted the inhibitory effects of Staurosporine on cell number.

In order to understand whether Magmas over-expression affects cell cycle progression, the latter was evaluated in control GH4C1 cells as well as in GH4C1-M-DD cells, treated without or with 200 nM Shield1. As shown in Figure 5B, treatment with Shield1 induced an accumulation of GH4C1-M-DD cells in S phase (+16% vs. control cells; P<0.05), an effect that was not observed in the absence of Shield1.

Finally, in order to quantify the percentage of cells undergoing apoptosis, we performed annexin V/pi staining. As shown in Figure 5C a strong increase in the percentage of cells undergoing apoptosis was observed in mock-transfected GH4C1 cells after treatment with Staurosporine (+ 30% vs. control untreated cells; P<0.05). On the other hand, in GH4C1-M-DD cells, the percentage of cells undergoing apoptosis after treatment with Staurosporine was lower (+21% vs. control; P<0.05 vs. Staurosporine-treated GH4C1-M-DD cells in the absence of Shield1).

### Magmas over-expression reduces caspase 3/7 activation by inhibiting cytochrome c mitochondrial release

**Figure 6:**
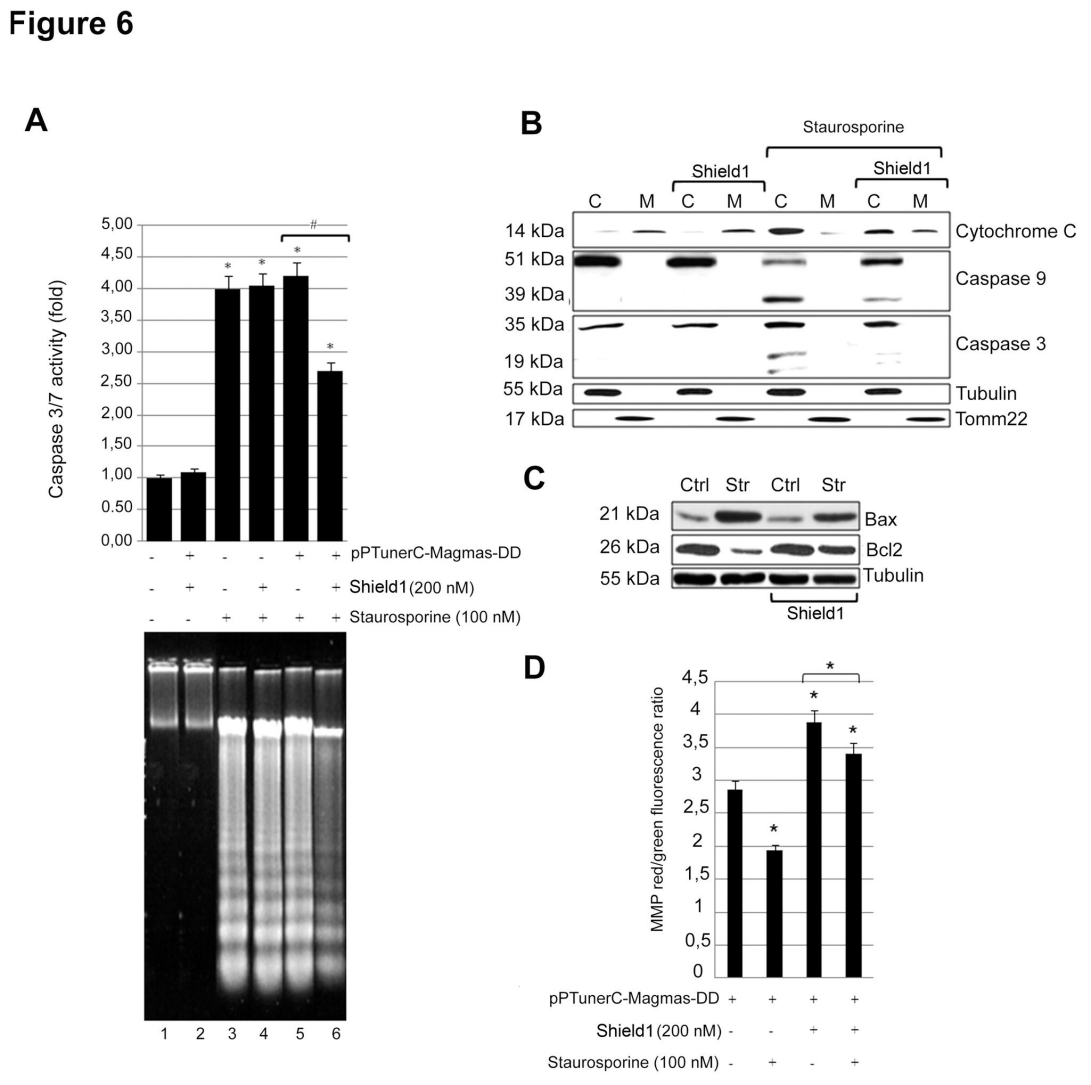
Magmas over-expression inhibits caspase activation and cytochrome c release. A) GH4C1 were transfected or not with the pPTunerC Magmas-DD vector, incubated in 96-well plates overnight and then treated for 48 h with or without 100 nM Staurosporine in the presence or in the absence of 200 nM Shield1. Caspase 3/7 activation (upper panel) was assessed as described in the Materials and Methods section. Data were evaluated in at least six independent experiments with eight replicates each and are expressed as the mean value ± SE fold induction vs. control cells. *P< 0.05 vs. control; #P<0.05 vs. GH4C1 cells treated with Staurosporine. DNA fragmentation analysis (lower panel) was assessed as described in the Materials and Methods section. 1= GH4C1 control cells; 2= GH4C1-MDD cells with 200 nM Shield1 3 = GH4C1 cells treated with 100 nM Staurosporine; 4 = GH4C1 cells treated with 100 nM Staurosporine and with 200 nM Shield1; 5 = GH4C1-M-DD cells treated with 100 nM Staurosporine; 6 = GH4C1-MDD cells treated with 100 nM Staurosporine and with 200 nM Shield1. B) GH4C1-M-DD cells were treated with or without 200 nM Shield1 in the absence or in the presence of 100 nM Staurosporine. Cytochrome c, total and cleaved caspase 9 and caspase 3 levels in cytoplasmic “C” and mitochondrial “M” fractions were analyzed by Western blotting as described in the Material and Methods section. Tubulin was used to normalize cytoplasmic fractions and TOMM22 to normalize mitochondrial fractions. A representative blot of three independent experiments is shown. C) GH4C1-M-DD cells were treated with or without 200 nM Shield1 for 12 hours and then for 48h in the absence or in the presence of 100 nM Staurosporine. Bax, and BCl2 protein levels were analyzed by Western blot as described in the Material and Methods section. The figure shows a representative blot among three independent experiments. Ctrl= control. Str= Staurosporine. (D) GH4C1 were transfected with the pPTunerC Magmas- DD vector and then incubated in 96-well plates for 48 h in culture medium supplemented with or without 100 nM Staurosporine, in the absence or in the presence of 200 nm Shield1. The JC-1 mitochondrial membrane potential assay was preformed (see material and methods). The data are expressed as the ratio between red and green fluorescence ± SE in at least three independent experiments. *P<0.05.

To further understand the mechanism by which Magmas counteracts the pro-apoptotic effects of Staurosporine, apoptotic mechanisms were further investigated. Figure 6A (upper panel) shows that Staurosporine potently induced apoptosis, as measured by caspase 3/7 activation, in GH4C1 cells both in the absence and in the presence of Shield1 (~3-fold vs. control cells; P<0.05). On the other hand, in GH4C1-M-DD cells the pro-apoptotic action of Staurosporine was significantly counteracted by Shield1 (-1.4-fold vs. GH4C1-M-DD cells in the absence of Shield1). DNA fragmentation analysis (Figure 6A lower panel) showed similar results. Indeed, Staurosporine strongly induced DNA fragmentation both in the absence and in the presence of Shield1 in GH4C1 cells. On the contrary, in GH4C1-M-DD cells Staurosporine-induced DNA fragmentation was greatly reduced in the presence of Shield1. These data demonstrate that Magmas over-expression counteracts the pro-apoptotic effects of Staurosporine by reducing caspase 3/7 activation, in keeping with the effects observed on cell proliferation.

Staurosporine triggers cytocrome c release from mitochondria, an early step of apoptosis, that is followed by caspase 9 activation, apoptosome assembly and, consecutively, caspase 3 activation [26-29]. Since Magmas is a mitochondrial protein, we investigated whether Magmas over-expression may affect cytochrome c release from mitochondria. To this aim, GH4C1-M-DD cells were exposed to Staurosporine for 12 h, in the presence or in the absence of 200 nM Shield1, and the amount of cytochrome c in cytoplasmic and mitochondrial protein fractions was analyzed. As shown in Figure 6B, in the absence of Staurosporine treatment, treatment with Shield1 did not modify cytochrome c translocation from the mitochondrial to the cytosolic fraction in GH4C1-M-DD cells, nor induced caspase 9 and 3 activation. On the contrary, under Staurosporine treatment cytochrome c translocated from the mitochondrial to the cytosolic fraction in GH4C1-M-DD cells in the absence of Shield1. On the other hand, in the presence of Shield1, under Staurosporine treatment cytochrome c translocation to the cytoplasm was reduced. In keeping with these results, Figure 6B shows that Staurosporine induced caspase 9 and 3 activation in GH4C1-M-DD cells in the absence of Shield1. In the presence of Shield1, caspase 9 and 3 activation by Staurosporine was greatly reduced. These results demonstrate that Magmas protects rat pituitary cells from pro-apoptotic stimuli by reducing Staurosporine-mediated cytocrome c mitochondrial release, consequently interfering with caspase activation. Moreover, to better characterize the mechanism by which Magmas reduces Caspase 3/7 activation we analyzed the expression of the pro-apoptotic protein Bax and the expression of the anti-apoptotic protein Bcl2. GH4C1-M-DD cells were treated with or without 200 nM Shield1 for 12 hours and then for 48h in the absence or in the presence of 100 nM Staurosporine. As shown in Figure 6C, in the absence of Staurosporine treatment, Shield1 did not modify either Bax nor Bcl2 expression. On the contrary, under Staurosporine treatment Bax expression increased in parallel with a decrease in Bcl2 protein levels compared to control cells. On the other hand, treatment with Shield1 reduced Staurosporine-induced Bax up-regulation and Bcl2 down-regulation.

To fully evaluate the consequences of Magmas over-expression on mitochondria, GH4C1-M-DD cells were treated with or without 100 nM Staurosporine and submitted to MMP evaluation As shown in Figure 6D, Staurosporine significantly (P<0.05) reduced MMP. On the contrary, treatment with Shield 1 significantly induced MMP (P<0.05), and blocked Staurosporine inhibitory effects.

## Discussion

In this study, we demonstrate that Magmas exerts protective effects towards apoptotic stimuli not only in a mouse ACTH-secreting pituitary adenoma cell line, but also in a rat GH/PRL secreting pituitary adenoma cell line. Furthermore, our data demonstrate that Magmas over-expression inhibits Staurosporine-induced apoptosis by hampering cytochrome c release from mitochondria in rat GH/PRL secreting pituitary adenoma cells, influencing Bax and Bcl2 modulation by pro-apoptotic stimuli. These results confirm and expand our previous findings obtained in a mouse ACTH-secreting pituitary adenoma cell line [11], indicating that the involvement of Magmas in the mechanisms that protect pituitary adenoma cells from Staurosporine-induced apoptosis are not restricted to the corticotroph lineage. Apoptosis is a crucial mechanism virtually active in all anterior pituitary cell types in rats [30], controlling pituitary cell number during pituitary development [31], estrous cycle [32], and regression from a hyperplastic state, such as pregnancy and lactation [33,34]. Programmed cell death may therefore be important for tissue remodelling and cellular networks organisation to ensure a suitable response to physiological stimuli.

We observed that only 2 out of the 4 investigated rat pituitary adenoma cell lines over-express Magmas as compared to normal rat pituitary. This finding is in keeping with a previous study [11], showing that not all of the human pituitary adenomas over-express Magmas and suggests that Magmas over-expression may represent a promoting rather than an initiating event in pituitary adenoma development. Comparing the whole pituitary to a pituitary adenoma tissue/cell line does not take into account that normal pituitary includes different cell types. However, the only available reference normal tissue to which the pituitary adenoma cell lines could be compared to is the whole pituitary, to date. A comparison could be performed with the normal pituitary cells by using immunohistochemistry or immunofluorescence, which, however, are not quantitative.

Our data also demonstrate that Magmas over-expression is associated with a significant increase in cell proliferation of rat pituitary adenoma cell lines, in agreement with previous findings [11]. In addition, it has been recently shown that Drosophila Schneider cells lacking Black-pearl, the yeast Magmas homolog in Drosophila, display a reduced proliferation rate and exhibit a 60% decrease in ATP levels [35]. We previously demonstrated that in mouse pituitary adenoma cells Magmas silencing prevents DNA synthesis and we here show that Magmas over-expression promotes an accumulation in the S-phase of the cell cycle in parallel with an increase in cell proliferation, measured both as viability (ATP levels) and cell number. In addition, Magmas over-expression does not affect basal apoptotic rate in rat pituitary cells, indicating that the increased cell proliferation is not due to a reduction in spontaneous programmed cell death. Indeed, Magmas protective effects towards apoptosis are apparent only in the presence of pro-apoptotic stimuli. Taken together, these results suggest that Magmas may be involved in promoting pituitary cell growth, activating survival pathways. However, we cannot exclude that the observed increase in intracellular ATP may be due to an enhanced mitochondrial function related to the oxidative chain. It has been demonstrated that, in yeast, Magmas, along with its co-chaperone Pam18, interacts with the respiratory chain [36], and that ATP5B, encoding for a subunit of mitochondrial ATP synthase, is significantly up-regulated in human pituitary adenomas as compared to control pituitary tissue [37]. On the other hand, we also provide evidence that the increase in ATP levels corresponds to an increased cell number, further supporting the role of Magmas in controlling cell proliferation.

Previous studies conducted in yeast models focussed on the role of Magmas protein in the TIM complex located on the mitochondrial inner membrane (4, 6, 8). And indeed, our data show that Magmas over-expression increases P and impairs Staurosporine inhibitory effects on this parameter. Some of the proteins contributing to TIM complex have been suggested as possibly involved in apoptotic mechanisms. It has been previously demonstrated that Tim23, the inner membrane translocase, plays a crucial role in caspase-independent apoptosis [38]. In addition, there is evidence that down-regulation of human Tim50, a component of the mitochondrial translocator, sensitizes human cell lines to death stimuli by inducing mitochondrial release of cytochrome c [39]. It has been suggested that, in these settings, the mitochondria are not able to import a large number of matrix proteins, with a consequent loss of mitochondrial integrity, permeabilization of the outer mitochondrial membrane, and release of the mitochondrial apoptotic proteins. A specular mechanisms could be hypothesised for Magmas over-expression, which might, on the opposite, increase mitochondrial integrity and protect the cells from pro-apoptotic stimuli. However, further studies are needed to demonstrate this hypothesis. Taken together, these evidences point to an involvement of the TIM complex in the regulation of apoptotic mechanisms.

Recent proteomic studies have shown that pituitary adenomas are characterized by mitochondrial dysfunction, involving also proteins of the inner mitochondrial membrane, to which Magmas belongs [37]. Pituitary adenoma mitochondrial dysfunction may be mirrored by the morphological changes reported in pituitary oncocytomas [40], in GH- and in PRL-secreting pituitary adenomas as well as in non functioning pituitary adenomas [41], where numerous and ultrastructurally abnormal mitochondria have been described. Furthermore, in rat prolactin-secreting pituitary adenoma primary cultures it has been shown that melatonin promotes apoptosis directly inducing mitochondrial damage [42], supporting the involvement of mitochondrial dysfunction in pituitary apoptotic processes. However, the extent and the specific consequences of mitochondrial dysfunction in pituitary adenoma development still remains to be fully elucidated.

## Conclusions

In summary in the present study we show that Magmas protects towards apoptotic stimuli also rat GH/PRL- secreting pituitary adenoma cells, beside mouse ACTH-secreting pituitary adenoma cells. In addition, we show that Magmas exerts its effects by regulating cytochrome c release mediated by apoptotic stimuli.
