# Correction: Magmas Overexpression Inhibits Staurosporine Induced Apoptosis in Rat Pituitary Adenoma Cell Lines

**DOI:** 10.1371/annotation/3af6faef-d942-4221-8d1a-47ce279e462b

**Published:** 2013-09-27

**Authors:** Federico Tagliati, Teresa Gagliano, Erica Gentilin, Mariella Minoia, Daniela Molè, Ettore C. delgi Uberti, Maria Chiara Zatelli

Due to a typesetting error, there was an error in the name of the sixth author.

The correct author name is: Ettore C. degli Uberti 

